# The Effects of Prenatal Alcohol Exposure on Executive Functioning

**Published:** 2001

**Authors:** Piyadasa W. Kodituwakku, Wendy Kalberg, Philip A. May

**Affiliations:** Piyadasa W. Kodituwakku, Ph.D., is a neuropsychologist, Wendy Kalberg, M.A., is an educational diagnostician, and Philip A. May, Ph.D., is a senior scientist and professor of sociology at the Center on Alcoholism, Substance Abuse, and Addictions, University of New Mexico, Albuquerque, New Mexico

**Keywords:** prenatal alcohol exposure, cognitive and memory disorder, brain damage, brain function, emotion, mood and affect disturbance, behavioral problem, neuroimaging

## Abstract

Converging evidence from various research areas indicates that people who have been exposed to alcohol prenatally may exhibit impairments on the performance of relatively complex and novel tasks. These tasks include tests designed to measure executive functioning (EF)—the ability to plan and guide behavior to achieve a goal in an efficient manner. EF can be categorized into two domains, cognition-based EF and emotion-related EF. People prenatally exposed to alcohol show impaired performance on tests assessing both domains. Moreover, one cognition-based and two emotion-related measures of EF appear to be reliable and stable predictors of behavioral problems in alcohol-affected people. A deficit in flexible recruitment of brain regions to do complex tasks may underlie the EF deficits in people prenatally exposed to alcohol.

The term “executive functioning” (EF) generally refers to cognitive functions involved in planning and guiding behavior in order to achieve a goal in an efficient manner. Impairments in EF have been found in patients with a wide range of neurodevelopmental disorders, including autism, attention deficit disorder, early treated phenylketonuria (PKU),^1^ and Fragile X Syndrome (Pennington et al. 1996). Studies found that children who had been exposed to alcohol prenatally may also be impaired on tasks measuring competencies associated with EF (Kodituwakku et al. 1995; Mattson et al. 1999) as well as on other cognitive functions (e.g., visual processing and memory functions). This article explores the relationship between prenatal alcohol exposure and deficits in EF. It first describes the cognitive skills subsumed under EF. It then summarizes the existing literature on EF in people who have been exposed prenatally to alcohol^2^ and discusses the usefulness of EF performance in defining a neurobehavioral profile related to prenatal alcohol exposure.

## Executive Functioning

The concept of EF refers to deliberate, or effortful, actions that involve various abilities, such as holding and manipulating information “in the head” (i.e. working memory) and focusing on one task at a time (i.e., inhibiting task-irrelevant habitual responses). Such deliberate actions can be contrasted with involuntary, or automatic, actions (for a more detailed discussion of this distinction, see the sidebar).

**Deliberate Versus Automatic Actions**

To achieve a preset goal, executive functioning (EF) requires deliberate, rather than automatic, actions. The difference between the two can be illustrated using a real-life situation. For example, when driving an automobile along a familiar route (e.g., to and from work), a driver typically accomplishes the task primarily in “automatic mode”—that is, without having to consciously think about where to stop at an intersection or where to make a turn. However, in certain situations in which an action is conducted based on automatic mode, deliberate attention is still required—for instance, if the driver’s familiar route includes an area designated as a school zone in which warning signs (e.g., flashing lights) are visible. In response to those lights, the driver automatically slows down and prepares to stop for children crossing the road. Furthermore, if a traffic light is situated in the middle of the school zone, the driver must stop when the light is red. When the light turns green, the driver must deliberately proceed at a slow speed and with caution until reaching the end of the school zone.

In this example, two processes enable the driver to resume driving automatically but also to deliberately maintain a slow speed: (1) using the “working memory,” which reminds the driver that he or she is driving in a school zone, thereby prompting the driver to consciously and deliberately respond to the situation appropriately, and (2) inhibiting the person’s “habitual response” of automatically accelerating to the “normal speed” (i.e., the legal speed limit outside the school zone) when seeing the green light. Both the effective use of working memory and the inhibition of inappropriate habitual responses are important components of EF. Consequently, a dysfunction in EF is characterized by actions that are inconsistent with the person’s goal (e.g., speeding in the school zone, rather than slowing down). Such actions are attributable to the effect of the immediate environment on a person’s behavior (e.g., when the driver automatically responds to the green light by accelerating to the speed limit posted outside of the school zone, rather than maintaining the slower speed required).

—P.W. Kodituwakku, W. Kalberg, and P.A. May

EF can further be divided into two categories. The original concept of EF referred to cognition-based actions, and researchers and clinicians have used a variety of cognitive tests requiring deliberate attention to formally assess this type of EF. Such tests measure problem solving, conceptual set shifting (described in the following paragraph), and rapid generation of verbal or nonverbal responses. Subsequently, some neuroscientists broadened the definition of EF to include another form of action selection that has been called emotion-related (Rolls et al. 1994), or affective (Dias et al. 1996) EF. Action selection at this level is based on rewards and punishments (i.e., positive and negative reinforcement) obtained in the past in similar situations. This emotion-related EF can be assessed using tests that measure the ability to modify behavior in response to changing reinforcement conditions.

### Assessing Cognition-Based and Emotion-Related EF

The distinction between cognition-based and emotion-related EF can be further illustrated by comparing two tests commonly used to assess EF. The Wisconsin Card Sorting Test (WCST) is used to evaluate cognition-based EF. In this test, the subject is asked to sort cards by a given dimension (e.g., the cards’ color) and then to shift attention to sorting them by a different dimension (e.g., the cards’ form) according to the examiner’s feedback. Thus, the WCST measures the subject’s ability to shift attention across different dimensions, a process formally known as conceptual, or extra-dimensional, set shifting.

Emotion-related EF can be assessed using the Visual Discrimination Reversal Test. In this test, the subject is required to learn stimulus–reward associations and to adjust responses when those associations are reversed. For example, the subject may be shown two images that appear one at a time on a computer screen. The subject then receives a “reward” (e.g., a pleasant sound) for responding to one of the images and a negative response (e.g., an unpleasant sound) for responding to the other image. Once the subject has learned this routine, the pattern of reinforcement changes without warning so that the rewarding image becomes nonrewarding and vice versa. The examiner then determines how quickly the subject adjusts to this reversal. Because this test measures the shifting of responses to two stimuli that vary in only one dimension (e.g., the pattern of the image), this type of response adjustment is called intra-dimensional set shifting. (Because the test measures the reversal of response-reward associations, some investigators also have used the term “affective shifts.”)

### Brain Regions Involved in EF

**Figure 1:**
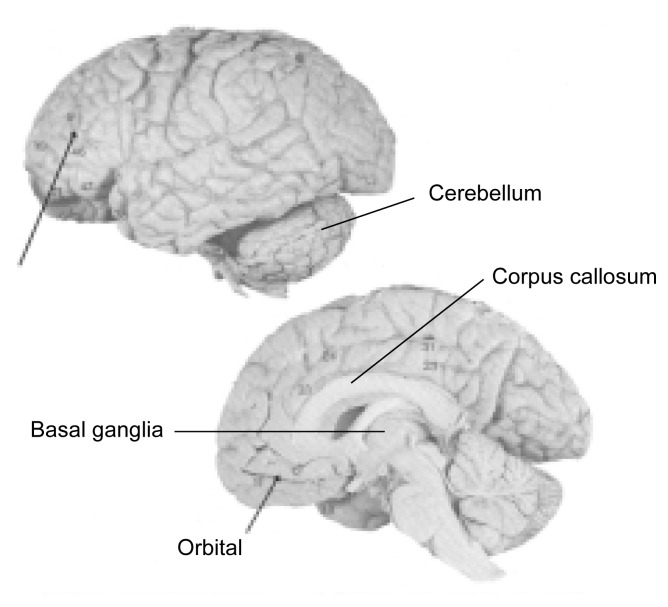
Images of the human brain showing two regions in the prefrontal cortex—the dorsolateral and the orbital—that are critical for cognition-based and emotion-related executive control functioning. Also indicated are other brain regions affected by prenatal alcohol exposure, including the cerebellum, basal ganglia, and corpus callosum. SOURCE: Modified from *Color Atlas of the Brain and Spinal Cord,* M.A. England and J. Wakely, 1991.

The brain regions that control EF can be identified by determining whether patients with damage in specific brain areas (regardless of whether that damage is alcohol related) show impaired performance on tasks assessing EF. Such analyses indicated that cognition-based EF and emotion-related EF are controlled by different brain areas. For example, Damasio (1994) reported that patients with damage in the orbitofrontal cortex (see figure 1) performed poorly on an emotion-related decision-making task but completed the WCST, which assesses cognition-based EF, with ease. Similarly, Rolls and colleagues (1994) found that patients with orbitofrontal damage were impaired in the Visual Discrimination Reversal Test, which assesses emotion-related EF, yet exhibited normal performance on cognition-based EF tasks. Furthermore, both Damasio (1994) and Rolls and colleagues (1994) found that the performance of patients with orbitofrontal damage on these emotion-related tasks was associated with the patients’ social and other behavioral problems—that is, patients with greater impairment on those tasks exhibited greater social and other problems.

Animal studies have indicated that cognition-based EF may be controlled by brain areas in the lateral prefrontal cortex (see figure 1). Thus, lesions in the lateral prefrontal cortex but not in the orbitofrontal cortex impaired the performance of monkeys on a test of extra-dimensional set shifting that was an analogue of the WCST (Dias et al. 1996). In contrast, monkeys with orbitofrontal lesions but not monkeys with lateral prefrontal lesions were impaired in performing intra-dimensional shifts in a Visual Discrimination Reversal Test.

## Cognition-Based EF in People Prenatally Exposed to Alcohol

Research has demonstrated that prenatal alcohol exposure can lead to brain damage in various regions. Moreover, alcohol-affected people exhibit a variety of behavioral and cognitive impairments. Given that the developmental outcome of alcohol-affected people is dependent on a wide range of variables (e.g., the amount and frequency of alcohol exposure, maternal age, and the parents’ education) it is difficult to determine a threshold of alcohol consumption for such adverse effects. Some researchers have obtained evidence, however, that seven standard drinks^3^ per week may be the threshold for most sensitive behavioral measures, although this threshold does not apply to all women and babies (Jacobson and Jacobson 1994). Although moderate alcohol exposure (i.e., 7.0 to 13.9 drinks per week) may produce impairments of EF, no relationship has been found between the number of abnormal physical features associated with heavy prenatal alcohol exposure and the degree of EF deficits in affected people. In other words, people with full-blown fetal alcohol syndrome (FAS)—the most severe consequence of prenatal alcohol exposure—and alcohol-exposed people without FAS both exhibit EF deficits to the same degree (Kodituwakku et al. 2001).

This section presents research on the performance of alcohol-affected people in tests assessing cognition-based EF. These tests use a range of tasks that involve holding and manipulating cognitive (or emotionally insignificant) information in working memory, such as tests of cognitive planning and strategy development, conceptual set shifting, rapid generation of verbal or nonverbal responses according to specific rules, and the ability to solve new problems quickly and accurately (i.e., fluid intelligence).

### Cognitive Planning and Strategy Development

**Figure 2:**
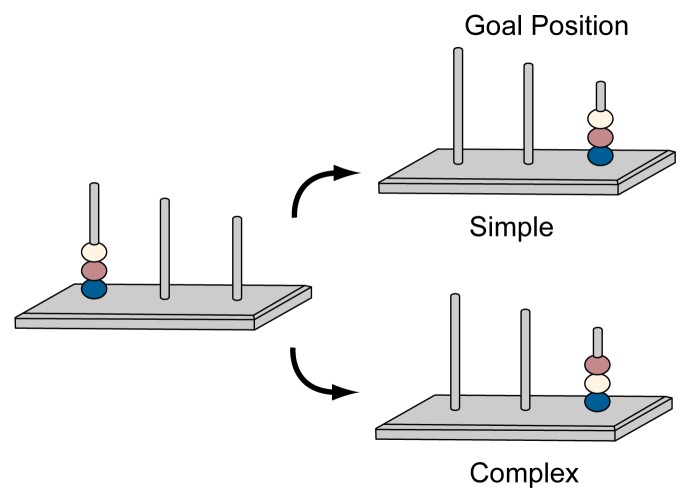
An illustration of two typical problems with different levels of complexity administered as part of the Progressive Planning Test (PPT). In both instances, the subject must move several beads from their initial positions to the goal position. When solving the problem, the subject can move only one bead at a time; once a bead has been moved from its initial position, it cannot be returned to that position. The problems shown here use three beads, each of a different color: yellow (Y), red (R), and blue (B). The first problem (labeled “simple”) has a relatively straightforward solution and, therefore, does not strain the subject’s working memory. The subject must move the beads as follows: the yellow, or Y, bead to the second (or 2) peg (denoted as Y to 2), then R to 2, B to 3, R to 3, and Y to 3. In contrast, the second problem requires greater mental manipulation by the subject, namely reversing the order of two beads (i.e., the Y bead and the R bead) when placing them in the second position before moving the B bead to the goal position. Thus, the subject must accomplish six steps to solve the problem: Y to 3, R to 2, Y to 2, B to 3, Y to 3, and R to 3.

Researchers have used a class of planning tests called look-ahead puzzles to assess cognitive planning skills in alcohol-exposed subjects. These tests require the subject to plan mentally several consecutive actions to solve a problem. Kodituwakku and colleagues (1995) used a test called the Progressive Planning Test (see figure 2) to assess planning skills in alcohol-exposed children with or without FAS. In this test, the participants are required to move three or four colored beads that are arranged in a specific order in an initial position on three stakes to create a series of prespecified new arrangements, or goal positions. The moves are subject to two rules: (1) only one bead can be moved at a time, and (2) once removed from its initial position, a bead must not be returned to that position. These rules constrain the “path” to the goal, increasing the working memory load involved in some problems.

In the study, the children had to solve a series of problems of increasing difficulty (see figure 2). The first set of problems (level 1 planning) was simple, with straightforward solutions that did not strain the working memory. In contrast, solutions to the second and third level problems involved greater mental manipulation, namely reversing the order of the beads before all beads could be moved to the goal position. Thus, the subject was required to organize a series of steps to solve the problems. The investigators found that prenatal alcohol exposure was associated with deficiencies in planning skills because the alcohol-exposed children had difficulty in solving the more difficult problems that involved mental manipulation.

Other investigators also observed deficiencies in the planning skills of alcohol-exposed people. For example, Mattson and colleagues (1999) found that alcohol-affected children were deficient in planning skills as measured by the California Tower Test—a lookahead puzzle like the Progressive Planning Test**—**and violated test rules more often than did a control group. Another test that examines planning skills and which has been shown to be challenging for patients with prefrontal cortical damage, is the Cognitive Estimation Test. In this test, subjects are asked to estimate quantities that are not readily available to them as part of established knowledge (e.g., the length of a dollar bill). To generate a reasonable answer to such a question, the subjects must rely on strategies that involve EF, specifically planning (Shallice and Evans 1978). Kopera-Frye and colleagues (1996) used this test for assessing planning skills in alcohol-exposed adolescents and adults. The investigators found that the alcohol-exposed participants tended to give unrealistic responses on this test, similar to patients with prefrontal damage.

Patients with deficits in EF also have been shown to have difficulty in learning new information, partly because of their inability to employ effective strategies (e.g., information organization) and their impulsivity. For example, Carmichael-Olson and colleagues (1998) found that on a spatial memory task, adolescents with FAS repeated the same error (i.e., committed perseverative errors) more often than control subjects and took longer to complete the task.

### Conceptual Set Shifting

As described previously, the WCST is a widely used test of extra-dimensional, or conceptual, set shifting. This test yields two primary measures of set shifting ability: (1) the number of perseverative errors—that is, sorting cards according to a previously correct dimension despite being told that the response now is wrong, and (2) the number of categories completed. Several studies found that alcohol-exposed children made more perseverative errors and, consequently, completed fewer categories than did control subjects on this test (Coles et al. 1997; Kodituwakku et al. 2001).

### Rapid Generation of Verbal Responses

Several tests can assess a person’s ability to generate rapid verbal or nonverbal responses (i.e., verbal and nonverbal fluency, respectively). In a commonly used test of verbal fluency, the subject is asked to produce words that begin with a specific letter (e.g., F or A) under certain constraints (e.g., without using proper nouns). The performance on this test is contrasted with that on a test measuring category fluency—the ability to generate words that pertain to a semantic category (e.g., animals). In a nonverbal fluency task, the subject is asked to rapidly generate designs according to specific rules. Patients with EF deficiencies have been shown to display a diminished ability on letter fluency tests (Benton 1968). However, little is known about letter and category fluency in alcohol-affected people. The study by Kodituwakku and colleagues (1995) indicated that alcohol-exposed children were more impaired in letter fluency than in category fluency. In a recent study, Schonfeld and colleagues (2001) found that children with substantial alcohol exposure were impaired in both verbal and nonverbal fluency.

### Fluid Intelligence

Researchers have observed that people’s performance on cognition-based EF tasks is similar to their performance on a range of fluid intelligence tests, which measure a person’s ability to solve new problems quickly and accurately. Both of these types of tests require the ability to hold and manipulate information in the working memory. A recent study using an imaging method called positron emission tomography (PET) indicated that a test of fluid intelligence activated a brain region—the lateral frontal lobe—which also is important to the abilities involved in cognitive EF tasks (Duncan et al. 2000), further supporting the notion that EF and fluid intelligence are related. Preliminary evidence suggests that alcohol-exposed children have more difficulty with fluid intelligence tests (e.g., Raven Progressive Matrices) than with crystallized intelligence tests—tasks that tap into established knowledge—such as the Peabody Picture Vocabulary Test (Kodituwakku et al. 1995).

## Emotion-Related EF in People Prenatally Exposed to Alcohol

As noted earlier, adults with damage to the orbitofrontal region of the cortex display a distinct pattern of impairments that involve emotion-related abilities, such as reversal of response-reward associations (Rolls et al. 1994). Because prenatal alcohol exposure can cause damage to that brain region or to a connected region, it is reasonable to speculate that alcohol-exposed people show deficits on tasks that are known to measure emotion-related EF.

To address this issue, Kodituwakku and colleagues (2001) used a modification of the Visual Discrimination Reversal Test developed by Rolls and colleagues (1994). This test consisted of two phases, a reversal learning condition and an extinction condition. In the reversal learning condition, the subject was instructed to respond to one of two patterns (i.e., fractal images) that appeared one at a time on the computer screen. One of the images was arbitrarily chosen to be “rewarding” and the subject was told that the goal of the test was to find out which image was rewarding (a “winner”) and which image was nonrewarding (a “loser”). The subject gained a point by responding to the rewarding image or by withholding a response to the non-rewarding image. Conversely, the subject lost a point by responding to the nonrewarding image or by failing to respond to the rewarding image. If the subject withheld a response to a stimulus, that image disappeared from the screen after 7 seconds, regardless of whether it was rewarding or not, and the subject received immediate feedback. For example, a correct response was followed immediately by a pleasant rising tone and the simultaneous on-screen appearance of the message “Congratulations, you have won a point.”

Similarly, an incorrect response was followed by an unpleasant short tone and the message, “Sorry, you have lost a point.” When the subject reached a certain learning criterion, which was 9 correct responses in a block of 10 images, the reinforcement contingencies changed without warning. The rewarding image became nonrewarding and vice versa. If the learning criterion was reached again, further reversals occurred up to a maximum of three reversals. The experiment was discontinued upon the subject completing three reversals or reaching the 100^th^ trial, whichever came first.

The term “extinction” refers to the gradual reduction of responses when no further reward is given. In the extinction condition of the test, the investigators used two new fractal images. The test began with the same instructions as given in the reversal condition. However, after the subject reached the learning criterion, responses to both images became nonrewarding. Thus, the subject could earn points only by withholding responses to both images. This part of the experiment was discontinued after the subject reached the criterion of extinction and withheld responses on 9 of 10 trials or had completed 30 trials after the initial learning phase.

The results showed that compared with control subjects, alcohol-exposed children and adolescents performing the reversal learning test were slower to reach the learning criterion despite receiving multiple practice trials, and they completed fewer reversals.

Importantly, this group difference in reversal learning became statistically significant after adjusting for the number of categories completed on the WCST. This means that when the performance of alcohol-exposed and control subjects with similar performances on the WCST was compared, alcohol-exposed subjects had significantly worse performance on the reversal learning test than did the control subjects. This result suggests that the reversal learning test and the WCST measure relatively independent functions.

With respect to the extinction condition of the test, greater variability in results existed in the alcohol-exposed group than in the control group. This means that even though the alcohol-exposed and control groups overall showed extinction after comparable numbers of trials, the alcohol-exposed group exhibited greater variability, with some subjects requiring a minimum number of trials and other subjects requiring a significantly greater number of trials.

## Working Memory and Response Inhibition in Alcohol-Exposed People

As mentioned previously, working memory and the inhibition of task-irrelevant responses are two fundamental processes that underlie both types of EF. Accordingly, it is reasonable to ask whether alcohol-exposed people exhibit deficiencies in these fundamental processes. Only limited research has directly evaluated the two processes in alcohol-affected children. Kodituwakku and colleagues (1995) detected no significant impairments in the alcohol-affected group on two widely used measures of working memory (i.e., delayed response tests and the Self-Ordered Pointing Test). Nevertheless, the alcohol-affected group demonstrated marked difficulty on relatively complex tasks that involve holding and manipulating information in working memory. Similarly, some researchers have failed to find evidence for response-inhibition deficits in alcohol-exposed children on certain neurocognitive measures (Kodituwakku et al. 1995; Coles et al. 1997). Mattson and colleagues (1999), however, have reported deficient performance of alcohol-exposed children on a measure of response inhibition called the California Stroop Test. Therefore, the presence and potential role of impairments in working memory and response inhibition in alcohol-exposed people requires further investigation.

## Behavioral Problems in Alcohol-Exposed People

A growing literature has reported that alcohol-exposed people display marked behavioral problems, particularly social deficits (Kelly et al. 2000). These include difficulty in understanding the social consequences of behavior and inappropriate interactions. To explore the relationship between EF deficits and behavioral deficiencies, Kodituwakku and colleagues (2001) used two parent-rated questionnaires—the Personal Behavior Checklist–36^4^ (Streissguth et al. 1998) and the Children’s Executive Functioning Inventory (CEFI)^5^—to assess behavior problems in alcohol-exposed and control children and adolescents. In addition, the investigators conducted several tests of cognition-based and emotion-related EF with the children. This analysis found that alcohol-exposed subjects exhibited significantly more behavioral problems than did the control subjects. Notably, the study also found that one measure of cognition-based EF—the number of perseverative errors on the WCST—and two measures of emotion-related EF—the number of omission errors and the variability in extinction—were reliable predictors of behavioral problems in the participants. Thus, deficient skills in both cognition-based and emotion-related EF were associated with behavioral problems of alcohol-exposed children. This finding is not surprising, however, given the multifaceted nature of the behavioral problems of alcohol-exposed people.

## Neural Correlates of EF Deficits in Alcohol-Exposed People

Neuropathological and neuroimaging studies have found evidence of structural brain damage in people with substantial prenatal alcohol exposure. For example, autopsy studies of children with FAS have found a wide range of neuropathology, including the absence or imperfect development of the corpus callosum (see figure 1) and disorganization of various cortical regions (Clarren 1986). Neuroimaging studies using magnetic resonance imaging (MRI) also revealed abnormalities in several brain regions of alcohol-exposed children, specifically in the basal ganglia, the corpus callosum, and parts of the cerebellum (Roebuck et al. 1998; for more information, see the article by Mattson and collegues, pp. 185–191, in this issue). However, these analyses detected no gross abnormalities in the frontal lobes of alcohol-affected children, which would have been expected based on studies linking EF deficits to brain damage in non-alcohol-exposed people. Accordingly, it remains unclear what brain abnormalities account for the deficits in EF in people with prenatal alcohol exposure.

Functional neuroimaging techniques (e.g., functional MRI), which can assess the activity of different brain areas while the subject performs various tasks, have shown that the brain recruits multiple regions to perform complex tasks that alcohol-exposed people find challenging (Carpenter et al. 2000). Accordingly, one can hypothesize that the EF deficits in alcohol-affected people likely are associated with a deficit in the efficient recruitment of brain regions required to do a task. The finding that alcohol-exposed infants process information slowly (Jacobson 1998) is consistent with this hypothesis. Abnormalities in specific brain regions, such as the basal ganglia, and in the connectivity among brain areas may contribute to such inefficient recruitment, as has been suggested for other mental disorders. For example, abnormal connectivity among brain regions has been proposed as a possible mechanism underlying frontal lobe dysfunction in patients with schizophrenia (Weinberger and Berman 1996). Neuroimaging studies of alcohol-exposed people have revealed evidence for disproportionately reduced white matter volumes (Archibald et al. 2001) and abnormalities of the corpus callosum, suggesting that the “neural infrastructure” required for information processing is compromised.

## Conclusions

Evidence obtained in studies using a variety of tests suggests that people who have been prenatally exposed to alcohol have deficient skills in both cognition-based and emotion-related EF. Such EF deficits may develop even in children of mothers who drank moderately (i.e., 7.0 to 13.9 standard drinks per week) during pregnancy; furthermore, alcohol-affected people with or without the characteristics of FAS have been found to exhibit comparable degrees of impairment on the performance of EF tasks. Some variables associated with conceptual and emotional set shifting appear to be reliable and stable predictors of behavioral problems as measured by parent-rated questionnaires. Accordingly, an improved understanding of the mechanisms underlying the EF deficits as well as of their neural correlates can have significant implications for the development of intervention methods for people with prenatal alcohol exposure.

## Footnotes

1 For a definition of this and other technical terms used in this article, see the glossary, p. 197.

2 Unless otherwise indicated, for the remainder of this article the terms “alcohol-exposed people” or “alcohol-affected people” will be used to refer to people who were exposed to alcohol prenatally rather than as adolescents or adults.

3 A standard drink is defined as 12 fluid ounces of beer, 5 fluid ounces of wine, or 1.5 fluid ounces of 80-proof distilled spirits, each of which contains approximately 0.5 fluid ounces of pure alcohol.

4 The Personal Behavior Checklist–36 consists of 36 items pertaining to several areas of functioning, such as social skills and interactions as well as communication and speech.

5 The experimental version of the CEFI includes 99 items designed to measure behavioral problems related to social appropriateness, inhibition, problem solving, initiative, and motor planning.

## GLOSSARY

Basal ganglia: A collection of masses of gray matter located deep within the brain that participate in emotion, cognition, and the regulation of motor performance.
Cerebellum: A small structure located at the back and base of the brain whose main functions are to control muscular activity and maintain balance.
Corpus callosum: A large fiber bundle connecting the areas of the two hemispheres.
Cortex: The outer layer of gray matter covering the brain; contains areas for processing sensory information, controlling motor functions, speech, memory, higher cognitive functions, emotions, and behavior.
Extinction: The “unlearning” of previously learned behavior when *reinforcement* is withheld.
Fragile X syndrome: A genetic condition caused by an unusual fragile site on one of the gender-determining chromosomes (i.e., the X chromosome); the most common inherited cause of mental retardation.
Phenylketonuria (PKU): A hereditary disorder characterized by the body’s inability to convert the amino acid phenylalanine to the amino acid tyrosine. Unless treated, phenylalanine accumulation causes brain damage.
Reinforcement: A procedure in which a response is followed by a rewarding event, such as the removal of a painful stimulus or receipt of an emotionally positive stimulus.
White matter: Areas of the nervous system rich in axons (thin elongated processes extending from nerve cell bodies) that are covered with glial cells. The glial cells, which provide insulation for the axons, are composed of a fatty substance that gives them a white appearance.
Working memory: The memory system involved in the temporary storage and manipulation of information when a person executes complex tasks, such as reasoning and comprehension.
